# Hydrogels for Exosome Delivery in Biomedical Applications

**DOI:** 10.3390/gels8060328

**Published:** 2022-05-24

**Authors:** Yaxin Xie, Qiuyue Guan, Jiusi Guo, Yilin Chen, Yijia Yin, Xianglong Han

**Affiliations:** 1 State Key Laboratory of Oral Diseases, Department of Orthodontics, National Clinical Research Center for Oral Diseases, West China Hospital of Stomatology, Sichuan University, Chengdu 610041, China; ashley_xieyaxin@163.com (Y.X.); jiusiguo@163.com (J.G.); dentistchenyl@163.com (Y.C.); yvonnist@163.com (Y.Y.); 2 Department of Geriatrics, People’s Hospital of Sichuan Province, Chengdu 610041, China; qiuyueguan@126.com

**Keywords:** composite hydrogel, exosome, biomedical engineering

## Abstract

Hydrogels, which are hydrophilic polymer networks, have attracted great attention, and significant advances in their biological and biomedical applications, such as for drug delivery, tissue engineering, and models for medical studies, have been made. Due to their similarity in physiological structure, hydrogels are highly compatible with extracellular matrices and biological tissues and can be used as both carriers and matrices to encapsulate cellular secretions. As small extracellular vesicles secreted by nearly all mammalian cells to mediate cell–cell interactions, exosomes play very important roles in therapeutic approaches and disease diagnosis. To maintain their biological activity and achieve controlled release, a strategy that embeds exosomes in hydrogels as a composite system has been focused on in recent studies. Therefore, this review aims to provide a thorough overview of the use of composite hydrogels for embedding exosomes in medical applications, including the resources for making hydrogels and the properties of hydrogels, and strategies for their combination with exosomes.

## 1. Introduction

Hydrogels are three-dimensional macromolecular polymeric networks composed of hydrophilic polymer chains. They can generally be divided into three categories according to their origin: natural, synthetic, and hybrid. Hydrogels are degradable, with a high affinity for water, and can be fabricated under physiological conditions, resulting in excellent biocompatibility [1]. They can be formed chemically and/or physically upon initiation with crosslinking agents and produced with a certain viscosity and elasticity. The innovation of Wichterle and Lim pioneered a new approach to applying crosslinked hydroxyethyl methacrylate (HEMA) hydrogels as biomaterials in 1960 [2]. In the two decades following this discovery, Lim and Sun demonstrated calcium alginate hydrogels with applications in cell encapsulation [3]. It is not surprising that hydrogels, having mechanical and structural properties similar to those of many tissues and the extracellular matrix (ECM), have attracted great attention, and significant progress has been made in designing, synthesizing, and using these materials for many biological and biomedical applications [4].

Exosomes are small, single-membrane, secreted extracellular vesicles (EVs), enriched in certain proteins, nucleic acids, and lipids. Budding at both the plasma and endosomal membranes of all the mammalian cell types studied to date, they are produced to remodel the ECM and deliver signals and functional macromolecules to adjacent cells. Numerous surface molecules on exosomes enable them to be internalized via endocytosis by recipient cells, playing an important role in regulating cell–cell communication [5]. Therefore, the study of exosomes in the pathology of various diseases is an active area of research, and the exploration of therapeutic exosomes as delivery vesicles has offered new insights for clinical applications in recent years. However, the stability and retention of exosomes released in vivo are major hurdles, as they are rapidly cleared by the innate immune system or accumulate in the liver, spleen, and lungs via the blood circulation [6].

**Figure 1:**
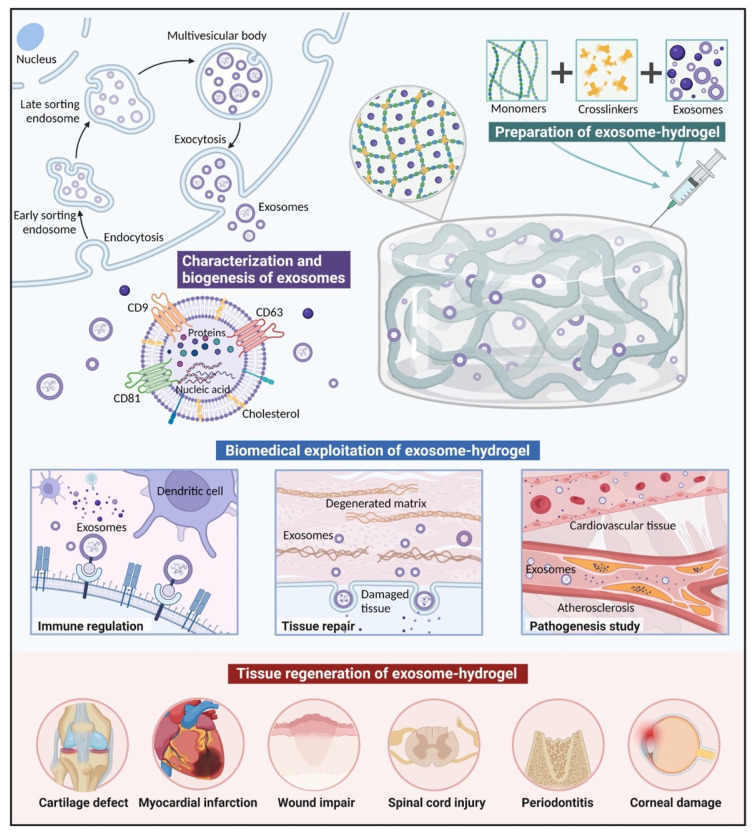
Hydrogels for exosome delivery in biomedical applications. Inward budding of the cellular plasma membrane results in the formation of endosomes, and the continuous inward invagination of the limiting membrane produces multivesicular bodies (MVBs). MVBs then fuse with the lysosome or plasma membrane, while the vesicles are released into the extracellular matrix to form exosomes. The secreted exosomes mainly contain proteins, nucleic acids, and lipids. The proteins contained in exosomes can be divided into two categories: one includes those commonly expressed in exosomes which can be used as markers (CD9, CD63, and CD81); the other includes the specific proteins from the parent cells. Hydrogels, as hydrophilic polymer networks, can encapsulate exosomes, overcoming the issue of low tissue retention and ensuring a controlled-release platform to localize their activity. Composite exosome–hydrogel systems have been applied in fields including tissue engineering and the study of pathogenesis. (Created with https://biorender.com/ accessed on 17 April 2022).

To overcome the rapid clearance and maintain the bioactivity of exosomes, hydrogels have been utilized to realize protection and controlled release by encapsulating these small vesicles. The excellent biodegradability of hydrogels allows them to be controlled by cell growth. Additionally, when they are used as scaffolds, exosomes can be loaded and released through their porous structure [7]. This review outlines the major applications of the hydrogel encapsulation of exosomes for physiological and pathological contexts, with a focus on the synthesis of, modification of, and exosome-loading strategies for hydrogels (Figure 1).

## 2. Hydrogels

Hydrogels, as hydrophilic polymer networks, absorb from 10–20% (an arbitrary lower limit) up to thousands of times their dry weights in water [2]. The high water content provides physical similarity to tissue, and gives the hydrogels excellent biocompatibility and the capability to easily encapsulate molecules [8]. The structural integrity of hydrogels depends on the crosslinks formed between the polymer chains, chemically and/or physically [9]. Naturally derived hydrogels are mostly formed by self-assembling physical crosslinks, including hydrogen bonds, van der Waals forces, and hydrophobic interactions, which cause macromolecules to fold and adopt well-defined structures and functionality [10]. Therefore, they can be synthesized in situ and used in injectable drug-delivery systems. Chemical crosslinking provides better stability because it allows substantially improved flexibility and spatiotemporal precision during gelation. The conventional mechanisms include radical polymerization, the chemical reaction of complementation groups, high-energy irradiation, and enzyme-enabled biochemistry, among others [11].

According to the different resources from which the polymers are sourced, hydrogels can be classified into natural, synthetic, and hybrid types. Recently, their network architectures, which can also be defined as conventional and unconventional polymer networks, such as interpenetrating and semi-interpenetrating polymer networks, have been extensively investigated. Concerning gel formation and drug release, a novel type of hydrogel capable of responding to a change in environmental conditions, such as temperature, pH, or the concentration of biomolecules, is the environment-sensitive hydrogel. As the design and preparation of hydrogels have been discussed in depth elsewhere, only a brief overview of common polymers is provided below [1,4,12].

### 2.1. Natural Hydrogels

Hydrogels derived from natural polymers tend to be highly compatible with biological tissues due to their similarity to the natural ECM or its components [13]. Therefore, the biodegradability and cell interactions in the tissue microenvironment of natural polymer networks mean that naturally derived hydrogels are widely used in tissue-engineering applications, and nearly all the hydrogels used for exosome encapsulation are based on naturally derived polymers [1]. The natural polymers for hydrogels can generally be divided into polysaccharides (alginate, hyaluronic acid, chitosan, agarose, and cellulose) and proteins (collagen, gelatin, and fibrin).

Alginate has been widely used as a scaffold in tissue engineering for cells, their encapsulation, and drug delivery; alginate is a linear polysaccharide copolymer of (1–4)-linked b-d-mannuronic acid (M) and a-l-guluronic acid (G) monomers and can be obtained from brown seaweeds and bacteria [14,15,16]. Alginate hydrogels are hypotoxic and easily available, while the dissociation of individual chains during gelation results in a loss of mechanical stiffness. Hyaluronic acid (HA) has been investigated for cell and molecule delivery, stem cell therapy, and tissue regeneration [17,18,19]. It is the simplest glycosaminoglycan (GAG), composed of a repeating disaccharide of (1–3)- and (1–4)-linked beta-D-glucuronic acid and N-acetyl-beta-D-glucosamine units, and is present in all mammals, especially in soft connective tissues [20,21]. HA hydrogels are nonimmunogenic, are biocompatible, and can be degraded by hyaluronidase for cell regulation. Chitosan is a linear polysaccharide, composed of β-(1–4)-linked D-glucosamine and N-acetyl-D-glucosamine, and obtained from arthropod exoskeletons [22]. Similar to naturally occurring GAGs, it has been applied in tissue engineering, showing excellent biocompatibility and biodegradability [23].

As the most abundant protein in animal bodies and the main component of the natural ECM, low-immunogenic collagen comprises three polypeptide chains wrapped around one another to form a three-stranded rope structure [24]. By introducing various chemical crosslinks, physical treatments, and other polymer modifications, collagen can be mechanically and stably enhanced, and is widely used in drug delivery and tissue reconstruction [25,26,27]. Gelatin is a single-stranded molecule naturally derived from breaking the triple-helix structure of collagen. Similar to collagen, gelatin requires covalent crosslinking, modifications, and interactions to further improve its physical properties [28,29]. GelMA hydrogels are hydrogels that are covalently crosslinked by introducing methacryloyl substituent groups to gelatin through photoinitiated radical polymerization [30]. A cargo-delivery platform can be created by mixing GelMA with nanoparticles such as laponite nanoclay to form a GelMA/nanoclay hydrogel with desirable combined mechanical and biological properties for specific biological applications [31]. Fibrin is a naturally derived polymer that is attractive for use in tissue sealants and adhesives for controlling bleeding in wound healing [32], as well as for scaffolds for tissue engineering [33,34]. It can be produced autologously from blood, thus possessing low antigenicity and being less likely to induce inflammatory responses [35].

However, the stability, mechanical properties, and cell adhesion of natural hydrogels need to be improved by extra crosslinking and modifications to realize specific biological and mechanical properties [13]. Covalent crosslinkers (e.g., glutaraldehyde and genipin) and physical treatments (e.g., UV irradiation and heating) have been applied to improve the mechanical properties of natural hydrogels [36,37,38,39]. A classic example of peptide modification is the introduction of the arginine–glycine–aspartic acid (RGD) sequence, which is used to enhance the cell-adhesion property [40].

### 2.2. Synthetic Hydrogels

Synthetic hydrogels can be fabricated with specific molecular weights, block structures, degradable linkages, and crosslinking modes to have tunable architectures at customized size scales and with controlled degradation rates. In addition, synthetic polymers are good in terms of cost, supply, and reproducible production. Examples of such synthetic materials discussed here are vinyl polymers (PHEMA and PVA), PEG, and polyesters (PLA).

Poly(hydroxyethyl methacrylate) (PHEMA) hydrogels can be prepared by the free-radical polymerization of HEMA. Copolymerization with acrylic or acrylamide monomers can achieve tunable swelling and mechanical properties for PHEMA hydrogels [41]. However, pure PHEMA requires extra biofunctional and bioactive motifs to realize cell adhesion and degradability in the tissue microenvironment [42]. PVA is mainly obtained from the partial or full hydrolysis of poly(vinyl acetate). Physically crosslinked PVA hydrogels exhibit high elasticity and fatigue resistance with low friction. PVA hydrogels, therefore, have been widely studied for cartilage tissue engineering [43]. Similar to PHEMA, pure PVA hydrogels need to be conjugated with several oligopeptide sequences to enhance their cellular interactions [44].

Hydrogels made from poly(ethylene glycol) (PEG) and the chemically similar poly(ethylene oxide) (PEO) are usually obtained from the polymerization of ethylene oxide [45]. Chemically crosslinked PEG hydrogels can be formed by photo-/UV-induced or radiation-induced free-radical polymerization with the modification of end chains with various chemical groups [46]. The physically crosslinked networks can also be generated by various motifs, which render the hydrogels reversible and stimulus-responsive [47]. Meanwhile, a triblock copolymer hydrogel has also been successfully manufactured and showed good performance for slow-release small EVs [48].

Poly(lactic acid) (PLA) is obtained from the ring-opening polymerization of lactide. The stability of PLA hydrogels can be improved via chemical crosslinking, such as photo-crosslinking to prevent autocatalytic decomposition [49]. Depending on the choice of lactide monomer, poly(_L_-lactic acid) (PLLA) and poly(D,_L_-lactic acid) (PDLLA) can be generated as stereoisomers, and result in differing stiffnesses for hydrogels encapsulating hMSCs [50].

The limitation of synthetic hydrogels is the lack of native tissue topography and structure. Ergo, hybrid hydrogels comprising both natural and synthetic materials have recently attracted increasing attention, with the biological moieties of natural materials being combined with the benefits of tunable synthetic materials [7]. They are defined as polymers composed of hundreds of chemically or physically crosslinked nanogels, or systems combined with different polymers and/or with nanoparticles. The structural similarity to the natural ECM, tunable viscoelasticity and mechanical properties, high water contents, and permeability for oxygen and essential nutrients make hybrid hydrogels good candidates for tissue-engineering scaffolds [51].

## 3. Exosomes

### 3.1. Characterization and Biogenesis of Exosomes

Nearly all types of mammalian cells secrete extracellular vesicles (EVs), including mesenchymal stem cells [52], immune cells [53], neuronal cells [54], endothelial cells [55], and cancer cells [56]. As determined by their biogenesis, EVs can be broadly divided into three categories: exosomes, microvesicles, and apoptotic bodies [57]. Exosomes originate from endosomes with a size range of 40 to 160 nm (average ~100 nm) in diameter [58]. The inward budding of the cellular plasma membrane results in the formation of endosomes, and the continuous inward invagination of the limiting membrane produces multivesicular bodies (MVBs) [59]. Therefore, they can selectively incorporate cytosolic contents, and transmembrane and peripheral proteins, which contributes to the heterogeneity of exosomes. MVBs may then fuse with lysosomes or the plasma membrane, while the vesicles released into the extracellular matrix form exosomes [60,61].

Exosomes mainly contain proteins, nucleic acids, and lipids; the proteins contained in exosomes can be divided into two categories. One comprises proteins commonly expressed in exosomes that can be used as markers to identify exosomes, such as the CD9, CD63, and CD81 tetraspanin proteins, as well as TSG101, Alix, flotillin, and Rab [62]. The other comprises specific proteins from exosomes from different sources. For example, exosomes from T cells can carry CD3 molecules [63]. A major feature of exosomes that can distinguish them from other biological vesicles is that they contain a large number of nucleic acids, including DNA, RNA, miRNA, and noncoding RNA [55,64,65,66]. Moreover, exosomes can be engineered to deliver diverse therapeutic payloads. Small RNAs (sRNAs), particularly microRNAs, are transferred to mediate cell-to-cell communication and deliver genetic information [67,68].

Since the above biogenesis of exosomes is physiologic behavior, large-scale production for clinical studies and commercialization requires a higher yield of exosomes. There are some strategies used to stimulate EV shedding and enhance yield that can also be explored for exosomes. Wang et al. found that exosome secretion by MSCs could be enhanced by N-methyldopamine and norepinephrine without altering their modulatory capacity [69]. Other strategies such as pH variations or low-oxygen conditions may also stimulate an increase in exosome production [70].

### 3.2. Isolation and Analyses of Exosomes

The heterogeneity of exosomes originates from their size, molecular content, functional impact, and cellular origin. Therefore, the isolation and detection of exosomes are necessary for their embedding in hydrogels [71]. A variety of conventional isolation and enrichment methods have been developed, including ultracentrifugation, gradient ultracentrifugation, coprecipitation, size-exclusion chromatography, and field-flow fractionation. Ultracentrifugation is the current gold standard and most commonly used conventional approach for exosome isolation [72]. Sucrose-gradient centrifugation can further fractionate according to different vesicular densities and is more typically used to isolate exosomes. Coprecipitation is performed using commercial kits that rely on polymer coprecipitation, which are expensive for large-scale usage and lack specificity for exosomes [71]. Size-exclusion chromatography and field-flow fractionation separate exosomes and other molecules based on their sizes and molecular weights [73,74]. Compared to conventional methods, various new enrichment methods such as microfluidic filtering, contact-free sorting, and immunoaffinity enrichment have been developed to improve the isolation efficiency and specificity [75,76,77,78].

Since the enrichment methods are mainly based on the size, structure, and capture of some of the membrane proteins of exosomes, it is necessary to study exosomes by physical, chemical, and biological characterization to distinguish them from other vesicles and macromolecular protein complexes. Scanning electron microscopy (SEM) and transmission electron microscopy (TEM) are widely used to determine the morphology and structure of exosomes [79]. Dynamic light scattering (DLS) and nanoparticle-tracking analysis (NTA) are still attractive techniques for measuring the concentrations and size distributions of exosomes [80,81]. Conventional methods for the detection of exosomal proteins include Western blotting, enzyme-linked immunosorbent assay (ELISA), mass spectrometry, and flow cytometry [82,83,84], while novel methods include micro-nuclear magnetic resonance (µNMR) and exosome sensors [85,86]. It has been found that exosomes are enriched with tetraspanins (CD9, CD63, and CD81), membrane trafficking proteins (RAB proteins and annexins), and MVB-related proteins (ALIX, TSG101, and clathrin) [87]. The nucleic acids of exosomes, as potential circulating biomarkers, and intercellular regulators can be amplified through polymerase chain reactions (PCRs) and sequenced [71].

Despite these developments, some questions remain for subpopulations of EVs lacking precise definitions. It is still difficult to distinguish exosomes from other small vesicles with confidence. According to the updated guidelines for studies of EVs, researchers are encouraged to consider the use of operational terms for EV subtypes that refer to physical characteristics, biochemical composition, or descriptions of conditions or cells of origin [88]. Therefore, many studies have regarded different types of EVs as an entire cargo to deliver a packaged set of bioactive components [89]. For the further understanding of EVs’ contents, single-EV analysis provides a benchmark by resolving EVs at a single-particle level [90]. Rogers et al. successfully detected EVs by using a single-EV microarray, which can assess EV proteins comprehensively and quantitatively [91].

### 3.3. Physiological Functions of Exosomes

Exosomes can be released under normal physiological conditions to regulate a range of biological processes. However, the precise roles of exosomes remain unclear due to the lack of physiological models in vitro and in vivo [90]. Ongoing experimental advances are likely to yield a thorough understanding of their heterogeneity and biological functions. The section below briefly discusses their main physiological functions.

1. Exosomes as mediators of intercellular communication.
  There are a variety of mechanisms that mediate cell–cell communication via exosomes. The phagocytosis-like uptake of exosomes by recipient cells enables them to transmit signals and molecules. Specific miRNA and protein cargoes in exosomes can contribute to tissue development and maintenance [92]. By directly fusing with the receptor cells, exosomes can exchange transmembrane proteins and lipids [93]. These properties mean that exosomes are involved in many physiological and pathological processes.
2. Exosomes as remodelers of the ECM.
  Cells can release exosomes into the ECM to manipulate its composition and function. Conversely, changes in the ECM affect cellular proliferation, migration, and organ morphogenesis. For example, exosomes can promote ECM synthesis by regulating matrix metalloproteinases (MMPs) [94], whereas some exosomes can inhibit the deposition of the ECM by suppressing collagen biosynthesis [95].
3. Exosomes as regulators of the immune response.
  Exosomes secreted by cells can modulate the immune response in various ways. Antigen-presenting cells can shed exosomes with the same cell-surface proteins such as MHC II and costimulatory signals [96]. An example of this is the release of exosomes containing bacterial mRNA by macrophages to activate the immune system [97]. MSC-derived exosomes can carry cytokines, miRNA, and other active molecules involved in proinflammatory and anti-inflammatory regulation [98].

## 4. Exosome-Loading Strategies

The stability and retention of exosomes are a major hurdle for clinical applications, as they are eliminated immediately by the immune system once injected in vivo [99]. Conventional delivery in cell-free exosome therapy includes intravenous, subcutaneous, and intraperitoneal injections. However, fluorescence imaging revealed that the majority of directly injected exosomes accumulated in various organs and tissues such as the liver and spleen [100]. Consequently, the method of administration should be optimized to achieve a high therapeutic efficacy and specificity, which requires delivering desirable exosomes to target tissues.

As the field rapidly evolves, biomaterials such as hydrogels allow exosomes to overcome the low tissue retention and ensure a controlled-release platform to localize their activity [7]. By embedding exosomes in a composite system, hydrogels play a dual role as carriers for cargo delivery and matrices for cellular interaction. Some of the first polymers used to synthesize hydrogels such as PHEMA and PEG are commonly used as cell culture materials. Much of the pioneering work with these hydrogels sought to elucidate the effects of the matrix stiffness on biological behavior [12]. However, these synthetic hydrogels are typically amorphous, homogeneous materials, considerably different from those of the native ECM. As progress has been made in 3D cell cultures, several strategies that permit cells and cellular molecules to spread and signal under physiological conditions have emerged. Hydrogels exhibiting passive hydrolytic degradation or cell-mediated enzymatic degradation have been considered, which enable the degradation rate of the matrices to be customized for the optimal release of the entrapped exosomes [4].

There are three common approaches for loading exosomes into a hydrogel matrix:

1. Polymers and exosomes are mixed and injected with crosslinkers in situ simultaneously.
  Exosomes are mixed with both polymers and crosslinkers simultaneously, and injected in situ with a dual-chamber syringe. After irradiation, ion exchanges, or environmental changes, polymerization can be achieved, inducing gelation [101]. In situ gelation can realize precise conformation to irregular cavities, and result in excellent integration and retention rates in the injection sites [102,103]. For example, entrapping effervescently generated CO_2_ bubbles can help to form highly interconnected porous networks in injectable hydrogels in vivo, which is conducive to cellular attachment, infiltration, proliferation, and ECM deposition [104].
2. Polymers and exosomes are incorporated before the addition of crosslinkers for gelation.
  Exosomes are combined with polymers followed by crosslinkers for gelation. For example, Qin utilized a composite matrix (thiolated hyaluronic acid, heparin, and gelatin) to encapsulate bone marrow stem cell (BMSC)-derived exosomes, followed by the addition of poly(ethylene glycol) diacrylate (PEGDA) as a crosslinker [105]. The combination based on covalent crosslinking improves the retention and release rates for the exosomes embedded in the polymers. A problem that cannot be ignored is that residual unreacted crosslinkers can be cytotoxic, drawing attention to optimizing the reaction conditions, such as the gelling temperature, and choosing alternative nontoxic crosslinkers such as genipin [37,106].
3. Polymers and crosslinkers are gelated before their physical combination with exosomes.
  This method involves dehydrating the already-swollen hydrogel and soaking it in a solution containing exosomes. Due to the super-water-absorbent and swelling properties of the hydrogel, the exosomes are absorbed into the porous structure [107]. On account of the weak physical incorporation of exosomes, the pore size is pivotal; exosomes may easily leak from large pores or have difficulty in entering through small pores.

## 5. Biomedical Exploitation of Exosomes Delivered in Hydrogels

Exosomes functioning in the delivery of functional cargos are currently an active research hotspot. The biological features of exosomes make them suitable as potential therapeutics for the diagnosis and treatment of several diseases. There are generally three approaches to obtaining exosomes with therapeutic and diagnostic potential. (1) Naturally derived exosomes (e.g., MSC-Exos) have been verified to be therapeutic by themselves [108]. (2) Engineering exosomes by transferring molecules such as microRNAs has achieved targeted applications [109]. (3) Exosome mimetics have been exploited as promising biomaterials [67,110]. Below, the emerging roles of exosomes in tissue repair, immune modulation, and the study of pathogenesis are discussed.

### 5.1. Tissue Repair

Of the many classes of biomaterials that have been used in tissue repair, hydrogels have been regarded as one of the most prominent and versatile for supporting most cellular behaviors and nutrient transport. Protected by them, cellular secretions can maintain their biological activity and undergo controlled release in pathological environments (Table 1).

#### 5.1.1. Bone and Cartilage Defects

Overwhelming evidence shows that the exogenous transport of miRNAs by exosomes can regulate osteogenic and angiogenic differentiation. An example of this is a study carried out by Mi et al., who created a cocktail therapy by transferring miR-26a-5p into endothelial cell-derived exosomes (EC-Exos) in an HA hydrogel. The EC-Exos^miR−26a−5p^ promoted osteogenic and osteoclast differentiation in mice with femoral fractures [109]. In another study, Hu et al. found that human umbilical cord MSC-derived small EVs (hUCMSC-sEVs) activated the PTEN/AKT signaling pathway by transferring miR-23a-3p when investigating the role and mechanism of cartilage regeneration [31]. Compared to increasing the specific miRNA in the target cells, the inhibition of miR-29a was verified to stimulate endogenous BMP/Smad signaling, which triggers subsequent osteogenic differentiation [67]. Therefore, the overexpression of miRNA can be an attractive method for improving the therapeutic effects. For example, miR-375 could be enriched in human adipose MSC (hASC)-derived exosomes by overexpressing the miRNA cargo in the parent cells [121].

**Figure 2:**
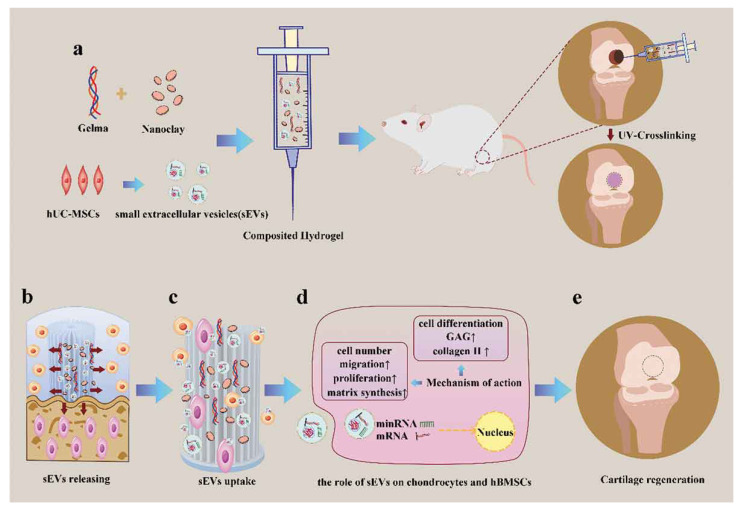
Schematic illustration of therapeutic sEVs released from a GelMA/nanoclay hydrogel for cartilage regeneration. (**a**) Preparation of a GelMA/nanoclay/sEV hydrogel and cartilage defect implantation. (**b**) Sustained release of sEVs with the degradation of the hydrogel. (**c**) Internalization of therapeutic sEVs by chondrocytes and hBMSCs. (**d**) The effect of the EVs on chondrocytes and hBMSCs. (**e**) Regeneration of a cartilage defect by the composite hydrogel. Copyright 2020, with permission from John Wiley and Sons [31].

Extensive research has shown that the essential properties of a bone and cartilage engineering scaffold are mechanical strength and a porous structure, to support the attachment and infiltration of osteogenic cells [122]. Hu et al. recently utilized an injectable and UV-crosslinked gelatin methacrylate (GelMA) to fabricate with nanoclay and achieved the sustained release of small EVs with the degradation of the hydrogel (Figure 2). The addition of laponite nanoclay significantly enhanced its ultimate strength for local administration in cartilage defects [31]. In addition to additives, 3D technology can also be applied to customize the shapes and sizes of porous scaffolds in accordance with bone defects. Fan et al. encapsulated umbilical MSC-derived exosomes (UMSC-Exos) in an HA hydrogel and combined them with 3D-printed nanohydroxyapatite/poly-ε-caprolactone (nHP) scaffolds [123]. Taken together, hydrogels can regulate extracellular matrix (ECM) formation, which provides a three-dimensional (3D) culture system for exosome secretion [89,124].

#### 5.1.2. Wound Repair

As a complicated biological process, wound healing consists of inflammation, proliferation, and remodeling [125]. The conventional treatment of chronic wounds includes regular wound debridement for stimulating skin regeneration and the protection of the wound using a specific dressing [126]. Recent interventions inspired by cell therapy approaches involve exosomes derived from MSCs, plasma, and cancer cells, while stem cell-derived exosomes are being developed for tissue recovery [68,127,128]. In a diabetes-impaired wound model, a wound dressing biomaterial was applied by combining antioxidant polyurethane (PUAO) for attenuating oxidative stress and adipose-derived stem cell (ADSC) exosomes for tissue remodeling [128]. Similarly, immobilizing ADSC-derived exosomes in a composite hydrogel that includes poly-ε-L-lysine (EPL), a natural cationic polypeptide from Streptomyces albulus, can help to realize antibacterial activity and adhesive ability [129]. Another study explored the feasibility of a composite hydrogel formed from silk fibroin (SF) and silk sericin (SS) due to the excellent mechanical properties of SF, and the cell-adhesion and biocompatibility properties of SS. After encapsulating and delivering UMSC-Exos, SF–SS hydrogels promoted wound healing and angiogenesis [130]. Additionally, the delivery of platelet-rich plasma exosomes in a composite chitosan–silk hydrogel sponge was found to upregulate collagen synthesis and deposition, as well as angiogenesis, at the wound site in diabetic rat models [127]. In addition, exosomes were enriched in miR-21, miR-23a, miR-125b, and miR-14, which can be blocked to reduce scar formation when they are laden in hydrogels [131]. Chitosan hydrogels functionalized with exosomes from synovium MSCs transduced to overexpress miR-126 promoted healing and angiogenesis in skin wounds [132].

#### 5.1.3. Cardiovascular Diseases

Ischemic myocardial infarction (MI) results from the severe blockage of blood arteries, which, in turn, interrupts nutrient supply. However, clinical treatments may lead to further myocardial ischemia/reperfusion injury [133]. New findings have triggered studies investigating the potential of utilizing MSC-derived EVs after MI to promote angiogenesis and restore cardiac function [117,134,135,136]. For example, Zou at al. elaborated an exo-anchoring conductive hydrogel enabling electrical conduction within the myocardial fibrotic area and promoting the synchronous contraction of the myocardium. In this study, an aniline tetramer (AT) was employed as a crosslinker, and the researchers endowed it with electroconductibility. The CP05 peptide was applied for its capability of binding to CD63 on the exosomal surface, to anchor and capture exosomes from human UC-MSCs [116]. Based on the intended application, hydrogels can be synthesized with different preparations. A notable application is to encapsulate EVs from induced pluripotent stem cells in a hydrogel patch and apply them directly onto the rat myocardium. The hydrogel patch enabled sustainable release, which protected the acutely injured heart against pathological hypertrophy [89].

#### 5.1.4. Spinal Cord Injury

Spinal cord injury (SCI) is among the most fatal diseases of the central nervous system, resulting in a temporary or permanent loss of sensation, movement, strength, and body functions [137]. To overcome the low cell survival resulting from the inhibitory environment at the lesion site, the local injection of exosomes protected by hydrogels is a promising therapeutic strategy. Li et al. improved the affinity of HA hydrogels and MSC-derived exosomes by a laminin modification, and successfully promoted spinal cord regeneration and the recovery of hindlimb motor function in vivo [119]. Surprisingly, plant (e.g., ginseng)-derived exosomes that can stimulate the neural differentiation of BMSCs have been demonstrated, and can be loaded in GelMA to fit the irregular shapes of injury defects [138]. The promotion of angiogenesis is beneficial for the regeneration of neuronal networks after SCI. Inspired by this, Luo et al. utilized a hybrid hydrogel system comprising GelMA, HA-NB, and a photoinitiator (LAP) to immobile exosomes from M2 macrophages. The hydrogel-mediated release system protected the exosomes from severe oxidative stress and inflammation [129].

#### 5.1.5. Other Diseases

In addition to the aforementioned applications, exosomes have also played important roles in periodontal, endometrial, and corneal repairs. In the context of periodontitis, the incorporation of dental pulp stem cell-derived exosomes and chitosan hydrogels repolarized macrophages and accelerated periodontal regeneration [108]. The dynamic coordination of adipose stem cell-derived exosomes and PEG hydrogels via Ag^+^–S resulted in outstanding injectable, self-healing, and antibacterial properties for endometrial and fertility restoration [113]. To effectively promote the repair of corneal damage, exosomes derived from MSCs were loaded in thermosensitive chitosan-based hydrogels [95].

### 5.2. Immune Regulation

Commonly, the adaptive immune response is regulated by antigen-presenting cells (APCs), such as dendritic cells (DCs), B cells, and macrophages, directly interacting with T cells and natural killer (NK) cells through cell-surface proteins [90]. Exosomes produced by APCs play an important role in the regulation of immunity, mediating immune stimulation or suppression, and driving inflammatory, autoimmune, and infectious disease pathology [96]. Inspired by dendritic cell-derived exosomes (DEXs), which improve cardiac function by activating CD4^+^ T cells in the spleen and lymph nodes [139], Zhang et al. encapsulated DEXs in a simple alginate hydrogel and injected the DEX-Gel into the MI model. The DEXs significantly upregulated the infiltration of Treg cells and M2 macrophages, which resulted in better wound remodeling, and preserved systolic function after MI. Furthermore, the combined application of the hydrogel provides physical support to the infarcted area [140].

MSCs confer regenerative effects in different tissue injuries, while in some cases, MSCs have been confirmed to secrete immunosuppressive cytokines and other factors, resulting in anti-inflammatory effects from stem cells [141]. Notably, the analysis of MSC-derived EVs revealed that they also have immunosuppressive therapeutic effects [142]. To harness EVs’ immunosuppressive properties, Fuhrmann et al. innovatively incorporated enzyme-loaded vesicles from MSCs into PVA hydrogels and applied this bioactive material for enzyme prodrug therapy. Once vesicles are released into the desired site, the injected nontoxic prodrugs are converted to anti-inflammatory drugs by enzymes [143]. The polarization of M2 macrophages, which can inhibit inflammation and induce tissue regeneration, has recently drawn great attention [108,109,144]. A classic cue is osteoimmunology, in which exosomes overexpressing miR-181 from human bone marrow-derived MSCs (hBM-MSCs) combined with a hydrogel were verified to significantly enhance osseointegration [144].

Tumor-derived EVs have been revealed to suppress tumor-specific and non-specific immune responses [96]. Metastatic melanoma releases a high level of exosomes carrying PD-L1 on their surfaces, which help in the evasion of immune surveillance. Based on how tumor cells suppress the immune system, Su et al. isolated exosomes from melanoma cells overexpressing PD-L1 to decrease T cell proliferation in a wound-healing model. The application of the thermoresponsive Pluronic F-127 hydrogel ensured that exosomes were released in a sustained manner [68].

### 5.3. Pathogenesis Study

Along with mediating physiological intercellular communication, exosomes also spread pathogenetic cargoes in diseases. Identifying the proteins and RNAs of exosomes can provide therapeutic targets. However, exosomal behavior can be dictated by the environment [4]. Therefore, hydrogels providing certain mechanical, structural, and compositional cues in the extracellular microenvironment are adopted as a novel strategy to recapitulate numerous physiologically relevant cell behaviors [145].

**Figure 3:**
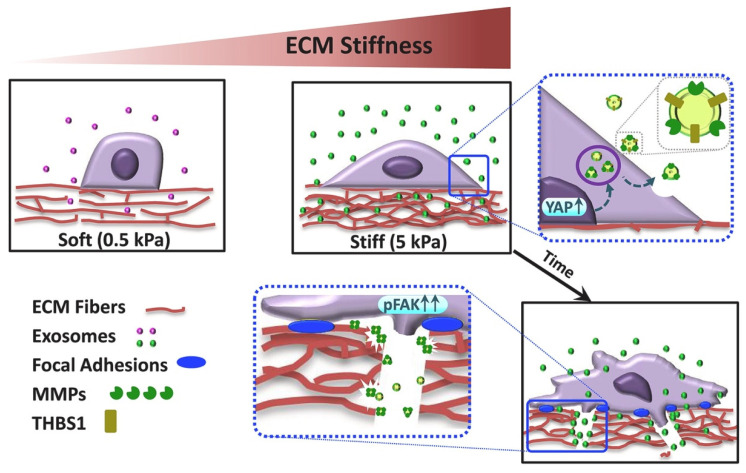
Proposed model of regulation of stiffness-dependent cancer invasiveness by stiffness-tuned exosomes. Breast cancer cells, in response to stiff substrates (5 kPa), secrete an excessive number of exosomes due to the activation of YAP/TAZ signaling. The stiffness-tuned exosomes confer an invasive, mesenchymal-like phenotype, accompanied by fostered focal adhesions and ECM remodeling. These changes are majorly elicited by exosomal THBS1 in concert with FAK and MMPs. Copyright 2021, with permission from Elsevier [146].

Tumor-derived exosomes can assist tumor growth and promote metastasis. To demonstrate the role of exosomes in ECM stiffness-triggered breast cancer invasiveness, Patwardhan et al. fabricated stiffness-tunable polyacrylamide (PA) gels as ECM mimics (Figure 3). Interestingly, stiff ECM cultures fostered exosome secretion by a series of changes in cell morphology, adhesion, and protrusion dynamics, which resulted in the invasion of breast cancer cells [146]. Aberrant cell behaviors can be induced by in vitro 2D culture, and the heterogeneity of exosomal behaviors also depends on the culture conditions [147]. Therefore, Millan et al. created 3D-engineered microtissues using the polysaccharides alginate and chitosan for the study of prostate cancer-derived EVs. Proteomics and RNA sequencing comparing 2D- and 3D-cultured cells revealed significantly differential expression of EV biomarkers. Some proteins known to be drivers of prostate cancer progression that were not detectable in the 2D conditions were enriched in the 3D cultures [148].

Exosomes from different cells such as endothelial cells and smooth muscle cells can contribute to atherosclerosis and cardiovascular disease when circulating in the blood [149,150]. In atherosclerosis-prone areas, EVs from smooth muscle cells (SMCs) and valvular interstitial cells (VICs) can cause a phospholipidic imbalance and, consequently, vascular and valvular calcification. Three-dimensional collagen hydrogels were utilized to produce a cardiovascular calcification model with which to observe the aggregation and microcalcification at the EV level [91]. Moreover, lesion macrophages can deliver exosomes that regulate vascular SMCs during the progression of atherosclerosis. In a study investigating the potential role of exosomes from nicotine-treated macrophages, Zhu et al. incorporated the above exosomes with chitosan hydrogels to stimulate release at the abdominal aorta [151].

## 6. Conclusions and Outlook

The recent development of hydrogels as biomaterials has been aided by progress in material science, polymer physics, preparation techniques, and biomimetic characteristics. Despite these advances, there remain many challenges and clinical needs for biological and biomedical applications. Secreted from parent cells, exosomes can become components of the ECM. Therefore, hydrogels, as loading and release systems for maintaining the bioactivity of exosomes, need to mimic the matrix. Conventional forms of optimization such as the tuning of the pore size, degradability, and compatibility may greatly improve the retention and release profiles of exosomes in vivo. For instance, 3D printing has been applied to improve the functional porosities, pore shapes, and geometries of hydrogel scaffolds [152]. Tunable release and prolonged delivery can also be achieved by introducing materials such as integrins into synthetic hydrogels [153].

Compared to enhancing biomaterial characteristics, how to deliver exosomes to target cells is more challenging because the interaction between biomaterials and cellular behaviors on a relevant timescale needs to be considered. Recent advances in prolonging the half-lives and increasing the purity of exosomes could be exploited in order to overcome this hurdle. Design strategies for composite gels that combine different types of polymeric components to obtain unique properties are, therefore, common. Further study needs to be undertaken regarding smart hydrogels, such as CRISPR gel, which can be tailored and render programmable gels from traditional materials, thus capable of providing spatiotemporally defined interactions with exosomes for clinical translation [154,155].

## Figures and Tables

**Table 1:** Advances in tissue regeneration via the hydrogel encapsulation of EVs.

Composite Hydrogel Type	Exosome Source	Release Kinetics	Therapeutic Application	Reference
GelMA/nanoclay hydrogel	hUCMSCs	90% in a month	Cartilage regeneration	[31]
HA hydrogel	ECs	80% in a week	Fracture repair	[109]
GMOCS hydrogel	BMSCs	80% in 2 weeks	Repair of growth plate injuries	[111]
PEO–PPO–PEO hydrogel	PRP	80% in 20 days	Subtalar osteoarthritis	[112]
Pluronic F-127 hydrogel	Melanoma cells	Release peaked at 24 h	Chronic wound repair	[68]
HA@MnO hydrogel	M2	Over 80% in 21 days	Repair of chronic diabetic wounds	[113]
Methylcellulose–chitosan hydrogel	PMSCs	Not mentioned	Severe wound healing	[114]
HA hydrogel	iPS-CPCs and iPS-MSCs	Lasting over 2 weeks	Cardiac remodeling after MI	[115]
AT-EHBPE/HA-SH/CP05 hydrogel	hUCMSCs	Not mentioned	MI and reperfusion injury	[116]
Gelatin–laponite nanocomposite hydrogel	hADSCs	Not mentioned	Repair of peri-infarct myocardium	[117]
PDNP–PELA hydrogel	ADSCs	92.5 ± 5.7% in 2 weeks	Erectile dysfunction treatment	[118]
Peptide-modified HA hydrogel	hPAMMSCs	80% in a week	Recovery from spinal cord injury	[119]
Chitosan hydrogel	DPSCs	80% in a week	Periodontitis	[108]
Fibrin hydrogel	Rat BMSCs	Left over 2 weeks	Tendon regeneration	[120]

hUCMSC (human umbilical cord mesenchymal stem cell); EC (endothelial cell); BMSC (bone marrow mesenchymal stem cell); OCS (chondroitin sulfate); GM (gelatin macryloyl); PRP (platelet-rich plasma); M2 (M2 macrophage); PMSC (placental mesenchymal stem cell); iPS (induced pluripotent stem cell); CPC (cardiac progenitor cell); MI (myocardial infarction); AT (aniline tetramer); EHBPE (epoxy macromer); HA-SH (thiolated hyaluronic acid); hADSC (human adipose-derived stem cell); PDNP (polydopamine nanoparticle); PELA (poly(ethylene glycol)poly(ε-caprolactone-co-lactide)); hPAMMSC (human placenta amniotic membrane mesenchymal stem cell); DPSC (dental pulp stem cell).

## Data Availability

The figures used and analyzed during the current review are available from the publisher and corresponding author on reasonable request.
